# Radiobiological effects of the alpha emitter Ra-223 on tumor cells

**DOI:** 10.1038/s41598-019-54884-7

**Published:** 2019-12-06

**Authors:** Kristina Bannik, Balázs Madas, Marco Jarzombek, Andreas Sutter, Gerhard Siemeister, Dominik Mumberg, Sabine Zitzmann-Kolbe

**Affiliations:** 1 Bayer AG, Pharmaceuticals Division, Berlin, Germany; 2 MTA Centre for Energy Research, Budapest, Hungary

**Keywords:** Radiotherapy, Apoptosis, Cell growth, Double-strand DNA breaks

## Abstract

Targeted alpha therapy is an emerging innovative approach for the treatment of advanced cancers, in which targeting agents deliver radionuclides directly to tumors and metastases. The biological effects of α-radiation are still not fully understood - partly due to the lack of sufficiently accurate research methods. The range of α-particles is <100 μm, and therefore, standard *in vitro* assays may underestimate α-radiation-specific radiation effects. In this report we focus on α-radiation-induced DNA lesions, DNA repair as well as cellular responses to DNA damage. Herein, we used Ra-223 to deliver α-particles to various tumor cells in a Transwell system. We evaluated the time and dose-dependent biological effects of α-radiation on several tumor cell lines by biological endpoints such as clonogenic survival, cell cycle distribution, comet assay, foci analysis for DNA damage, and calculated the absorbed dose by Monte-Carlo simulations. The radiobiological effects of Ra-223 in various tumor cell lines were evaluated using a novel *in vitro* assay designed to assess α-radiation-mediated effects. The α-radiation induced increasing levels of DNA double-strand breaks (DSBs) as detected by the formation of 53BP1 foci in a time- and dose-dependent manner in tumor cells. Short-term exposure (1–8 h) of different tumor cells to α-radiation was sufficient to double the number of cells in G_2_/M phase, reduced cell survival to 11–20% and also increased DNA fragmentation measured by tail intensity (from 1.4 to 3.9) dose-dependently. The α-particle component of Ra-223 radiation caused most of the Ra-223 radiation-induced biological effects such as DNA DSBs, cell cycle arrest and micronuclei formation, leading ultimately to cell death. The variable effects of α-radiation onto the different tumor cells demonstrated that tumor cells show diverse sensitivity towards damage caused by α-radiation. If these differences are caused by genetic alterations and if the sensitivity could be modulated by the use of DNA damage repair inhibitors remains a wide field for further investigations.

## Introduction

The main concept of targeted alpha therapy (TAT) is that a single α-particle with high linear energy transfer (LET) is sufficient to damage the cell nucleus, as cell death due to α-radiation is independent of oxygenation or active cell proliferation^1^. Radium-223 dichloride is a first-in-class in TAT and has been shown to prolong survival in patients with castration-resistant prostate cancer and symptomatic bone metastases without visceral metastases^2^. High-LET radiation, such as α-particles, is more efficient at producing complex DNA damage (also termed clustered DNA damage), than low-LET^3,4^. Although the therapeutic effect of α-particle treatment has been demonstrated preclinically, the interpretation of biological effects of α-radiation in tumor cells is not fully understood.

In order to design a successful TAT agent, several points have to be considered. First, the selection of the appropriate radionuclide depends on many requirements. Each radionuclide used in therapy is characterized by its own decay properties, including the decay energy (determining the alpha particle range in the tissue) and half-life ranging from several hours (Bi-212: t_1/2_ = 60.6 min, At-211: t_1/2_ = 7.2 h) to many days (Ra-223: t_1/2_ = 11.4 d, Th-227: t_1/2_ = 18.7 d). Second, the selection of most appropriate TAT molecules will also depend on the targeting antigen and it expression frequency, density, specificity and location (e.g. Prostate specific membrane antigen - PSMA, human epidermal growth factor receptor 2, c-erbB2 - HER2/neu, mesotelin –*MSLN*, B-lymphocyte antigen *CD20*) proposing the opportunity to adapt the features of the TAT agent to a particular type of cancer. And thirdly, important is the *in vivo* pharmacokinetics of targeting agent (a single chain bispecific antibody (*scFv*_*2*_), bispecific antibodies (*bsAb)*, fragments antibodies *(Fab)*, or a peptide), together with a conjugated chelator (DOTA, NOTA or HOPO)^5,6^. The physical half-life of the radionuclide should approximately match the biological half-life of the targeting agent. Having designed a successful TAT, a radiation dose is delivered to the tumor causing damage to the tumor cells. There are two approaches to measure the absorbed dose delivered by α-particles and to provide a basic for understanding the effects and efficacy of different radiation-based treatments: (i) using a Germanium detector directly^7^ and (ii) a theoretical method, which has been described by the Medical Internal Radionuclide Dose (MIRD) Committee^8,9^.

**Figure 1:**
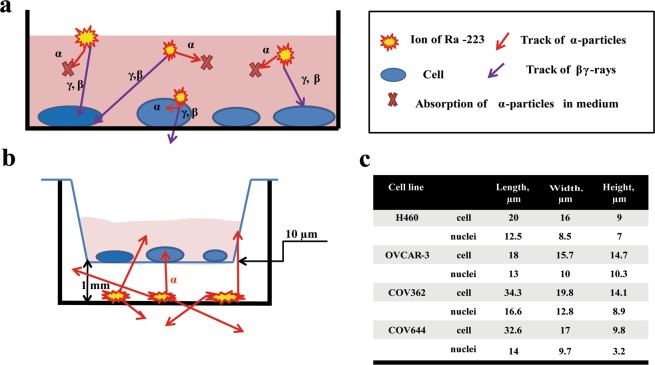
Schematic representation of the irradiation geometry. (**a**) The absorption of α-particles in medium. (**b**) The schematic pathway of α-particle from Ra-223. (**c**) The geometric data of several human cancer cell lines.

In order to gain further insights into the biological effects of α-radiation on tumor cells, the present study aimed to evaluate the time-dependent biological effects of α-radiation *in vitro* in various tumor cell lines by investigating radiobiological endpoints like DNA damage and its repair, cell cycle distribution and cell survival. As an initial step, we used the Transwell system (TW) to simplify the study design of delivering α-particles directly to cancer cells (Fig. 1). TW offers the opportunity to study several cell lines without having to take into account target expression, target recycling and compound kinetics. Radiation can be applied directly without delay and can be removed at will to allow time dependent studies of cellular effects. The cell size and the exact irradiation geometry were measured in order to quantify the absorbed doses from α-radiation in the cells using Monte-Carlo techniques based on fundamental physical principles.

## Materials and Methods

### Cell culture

Tumor cell lines were obtained from the American Type Culture Collection, the German Collection of Microorganisms and Cell Cultures, the European Collection of Authenticated Cell Cultures, or the National Cancer Institute. Authentication of the cell lines used was performed at the German Collection of Microorganisms and Cell Cultures via PCR-based DNA profiling of polymorphic short tandem repeats. Cells were propagated in DMEM medium (ES-2, COV644) or in RPMI-1640 medium (NCI-H460, 22Rv1, OVCAR-3, HCT116, A549, NCI-H1299) supplemented with 10% fetal bovine serum (ThermoFischer, MA) and 1% Antibiotic Antimycotic solution (Sigma). All cultures were incubated at humidified 37 °C in 5% CO_2_.

### Dosimetry

A Transwell system (TW) consists of a culture plate and an insert with the membrane. The cells were seeded onto the membrane of Transwell system of various sizes (#3450, #3460 or #3470, Corning). Growth of the cells on the insert membrane was tested and compared with growth on corresponding well plate. The exposure to α-particles was performed by coating the bottom of a TW with Ra-223. To achieve an even coating, Ra-223 (Xofigo, Bayer AG, Germany) in its ionic form was mixed with an aqueous 70% ethanol solution and was dried overnight. The cells were seeded 24 h prior to radiation and irradiated with α-particles from the bottom of the wells through the 10 µm mylar membrane. 95.3% of the emitted energy from Ra-223 is attributed to alpha emissions. Four α-particles are emitted in total per decay chain until a stable nuclide Pb-207 is formed. The absorbed doses in the cells and the hit distribution from the α-particles of all progeny from Ra-223 were calculated by Monte-Carlo simulations, the doses from β- and γ-radiation were neglected. The use activity is defined as the activity of Ra-223 divided by the area of the well’s surface was determined to be 1.32 kBq/cm^2^. The cumulative dose was calculated. Additionally, radioactivity and time of exposure are also given.

### Cell characteristics

The cell morphology of H460 was determined with live cells using an Operetta CLS high-content analysis system (non-confocal). Cell thickness measurement was performed by adding Hoechst 33342. The cell size data were used for the Monte Carlo simulations.

### Immunofluorescent staining

After irradiation, the cells on the TW insert membrane (#3460, Corning) were fixed with 4% paraformaldehyde for 15 min, washed with PBS, permeabilized (100 mM TrisCl pH 7.4, 50 mM EDTA, 0.5% Triton100 in H_2_O) and blocked (3% BSA, 0.1% Tween20, 4xSSC, 7.7 mM NaN_3_ in H_2_O). The cells on the membranes were incubated overnight at 4 °C with the primary antibodies anti-phospho histone γH2AX (Millipore, 05-636) and anti-53BP1 (Abcam, ab21083) in PBS. The cells were washed with PBS and incubated with a secondary antibody labeled with Alexa488 (A 2102, Invitrogen) or Cy3 (BA 1034-05, Boster) and DAPI 1 mg/ml (Thermo Scientific) for 90 min at room temperature. The membranes were cut manually and transferred onto slides, mounted with immu-mount and cover-slipped. The γH2AX and 53PB1 foci per nucleus were identified by eye and calculated manually using a Leica DMi8 fluorescent microscope (USA).

### Cell cycle distribution

The cells were plated into TW inserts (#3450, Corning) with a density of 0.2 × 10^6^ cells/well. After irradiation, the cells were detached with trypsin, washed with PBS and fixed with 70% ethanol overnight at −20 °C. For the cell cycle analyses, the cells were washed with PBS and treated with RNaseA (Sigma, R-4875, 5% in PBS) and stained with propidium iodide (PI) (Sigma, P-4170, 100 µg/ml in PBS) for 2 h at 4 °C. The samples were assessed for cell cycle distribution (cell cycle phases) using a FacsCalibur 3CS (Becton Dickinson, USA). The results were analyzed by using software CellQuest.

### Cell survival

The cells were plated into TW inserts (#3450, Corning) with a density of 0.2 × 10^6^ cells/well. On the following day the cells were irradiated with Ra-223 α-particles for different lengths of time (2, 4 and 8 h) to deliver increasing amounts of radiation doses. After irradiation, the cells were incubated for an additional 24 h. The cells were collected and counted again, before the cells were re-seeded into fresh medium in new 6-well plates in triplicate. Due to the broad difference in the plating efficiency of cancer cells and the different doses, the number of cells was adjusted for each cell line and dose, and then incubated for colony formation for 9 to 21 days, depending on the cell growth rate. A colony was defined as consisting of at least 50 cells. The colonies were fixed with 11% glutardialdehyde (Merck) for 20 min and stained with 10 times diluted crystal violet solution (Sigma). The colonies were counted with the naked eye without any magnification and plating efficiency and survival were calculated. The plating efficiency (PE) in percent was determined by calculating the number of colonies formed/number of cells seeded) × 100%. The survival fraction was determined by the number of colonies formed after treatment/number of cells seeded × PE.

### Comet assay

The cells were seeded into TW inserts (#3450, Corning) with a density of 0.15 × 10^6^ cell/well. Next day, HEPES buffer (10 mM) was added 10 min before irradiation. The cells were α-irradiated for 1 h. Some of TWs were irradiated on ice (4 °C) and other TWs in the incubator at 37 °C. After irradiation, the cells were immediately detached with trypsin into cold PBS. All steps for the comet assay were performed according to a standard procedure^10^. In brief, the cell suspension was mixed with 0.5% low melting agarose and transferred onto slides. After incubation in the lysis buffer and alkaline treatment in electrophoresis buffer, electrophoresis (20 min, 25 V and approximately 300 mA) and neutralization were performed. Subsequently, slides were dehydrated with ethanol. For evaluation, the cells were stained with propidium iodide (Sigma) on the day of analysis. The comets were evaluated by image analysis using the software comet IV (Perceptive Instruments, Haverhill, UK). Tail intensity (% tail DNA) was used as a primary assessment parameter. The comets were semi-automatically analyzed (duplicates, 50 cells per technical replicate).

### Dose determination

Absorbed doses and distribution of cell nucleus hits were simulated by Monte-Carlo methods for several human cancer cells. Figure 1b schematically shows the radiation geometry of a TW insert. The cells were seeded in a TW insert (upper well) on its round mylar membrane (biaxially-oriented polyethylene terephthalate) with a radius of 6 mm and a thickness of 10 μm. This TW insert is placed over the radiation source. The upper and lower wells are concentric and the distance between them is 1.016 mm. This means that α-particles have to cross air and mylar to reach the cells. If the α-particles are emitted in an angle, the length of the α-particle track both in the air and in the membrane will be larger (if the angle is too large, the α-particles will be absorbed by the plastic of the well). The general set up remains the same. Basic geometric data for several cancer lines were measured and applied for the absorbed doses calculation (Fig. 1c).

For Monte-Carlo simulations, we set up a Fortran code in CodeBlocks Interactive Development Environment. The number of α-decays was computed using the initial activity and the duration of exposure supposing that the four alpha-emitting radionuclides (Ra-223, Rn-219, Po-215, Bi-211) of the decay chain have the same activity and produce the same amount of α-particles. The location of radioactive decays was selected randomly using a uniform distribution over the area of the bottom well. The direction of movement was selected randomly in the 4π solid angle. The energy loss of each α-track in the air, in the mylar foil and in the cells was calculated using the Stopping and Range of Ions in Matter (SRIM) code^11^ selecting ICRU-104 Air Dry, ICRU-222 Mylar (Polyethylene Terephthalate), and Trachea (W&W) from the compound directory, respectively. In order to estimate the dose distribution at different depths in the media, the energy loss in 1-μm-thick layers was computed. For the α-particles having a positive kinetic energy after crossing the mylar membrane, the average doses absorbed by cell nuclei were estimated first by numerical integration of the dose distribution considering the volume of the nuclei in these layers. Absorbed doses in individual cell nuclei were also computed and average cell nucleus dose and its standard deviation was determined from this distribution.

### Statistical analysis

All experiments were performed at least two times. Numerical data were analyzed using ANOVA test with Tukey’s multiple comparisons test in the GraphPad Prism 7. Significance thresholds are indicated in legends (*p < 0.05; **p < 0.01; ****p < 0.0001).

## Results

### Absorbed dose determination

**Figure 2:**
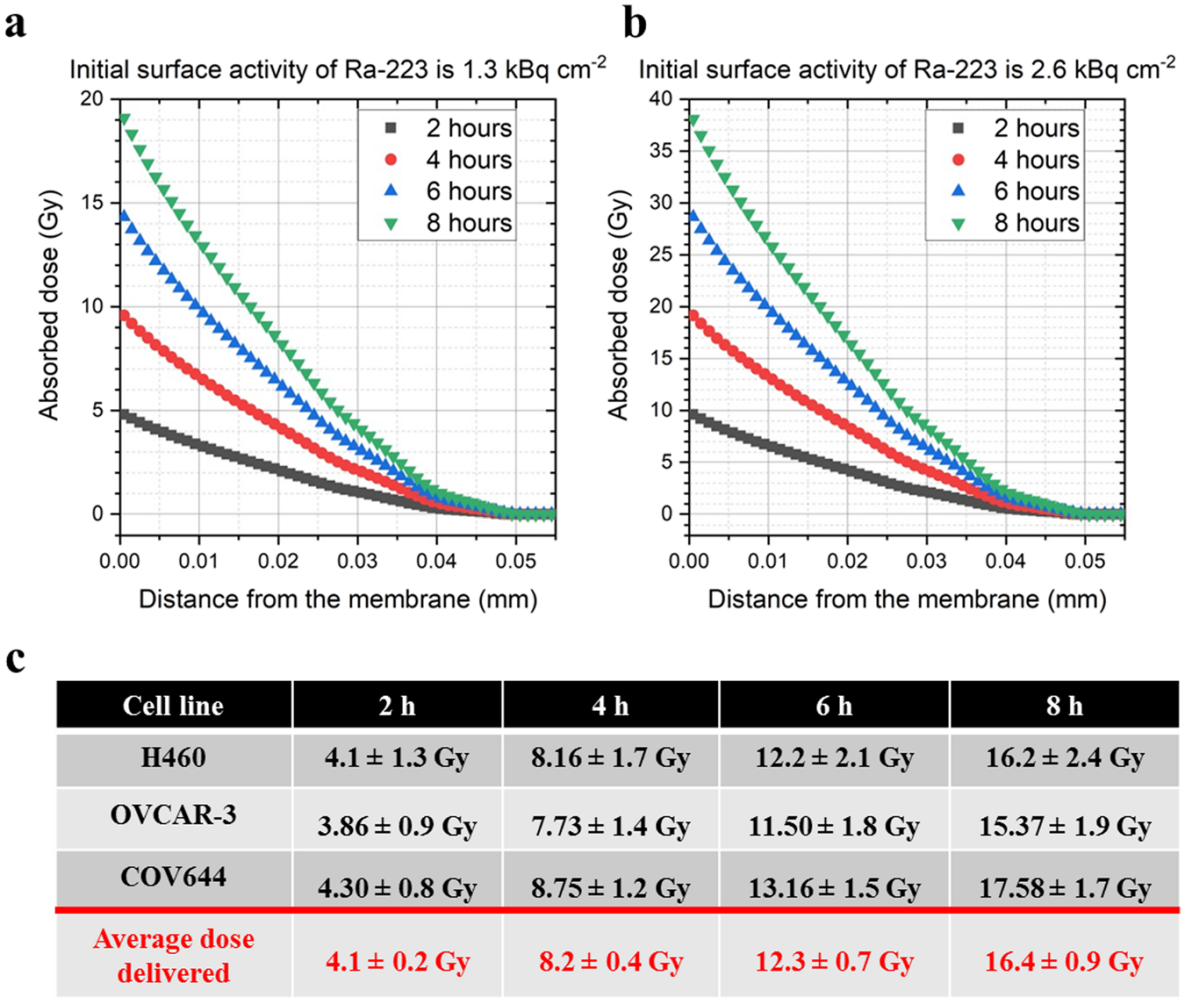
Absorbed dose in different layers on the medium/cells as a function of the distance from the mylar membrane with the initial activities of Ra-223 - 1.3 kBq/cm^2^ (**a**), 2.6 kBq/cm^2^ (**b**). Data points are plotted for the middle of the layers. (**c**) Table of absorbed dose in various tumor cells (mean ± SD).

Figure 2 shows the dose distribution for two different radiation does: 1.3 kBq/cm^2^ (Fig. 1a) and 2.6 kBq/cm^2^ (Fig. 2b) over fixed lengths of time (2, 4, 6 and 8 h) and by increasing the distance of the dose from the membrane through the media and cells incrementally by 1 µm, and clearly demonstrates a sharp decrease in absorbed dose as a function of depth (Fig. 2a). Furthermore, it provided an insight how slight changes in the thickness of the “shielding” (source, air, membrane, target) influenced the radiation doses. No α-particle could reach beyond the depth of 51 μm into the medium, and therefore absorbed dose is zero if the depth is higher. The average absorbed dose and its standard deviation in tri-axial ellipsoid-shaped cell nuclei located 1 µm above the membrane were computed for the individual cell nuclei. When the initial activity of Ra-223 was 1.3 kBq/cm^2^, the average dose delivered to H460 cells is 4.1 ± 1.3, 8.16 ± 1.7, 12.2 ± 2.1, and 16.2 ± 2.4 Gy upon exposure for 2, 4, 6, and 8 hours, respectively. As shown in Fig. 2c, the absorbed doses in other cell lines were quite similar. Therefore, we calculated the average dose delivered from all cell lines and used this value for all cell lines in this paper.

### Ra-223 damages primarily via α-radiation

**Figure 3:**
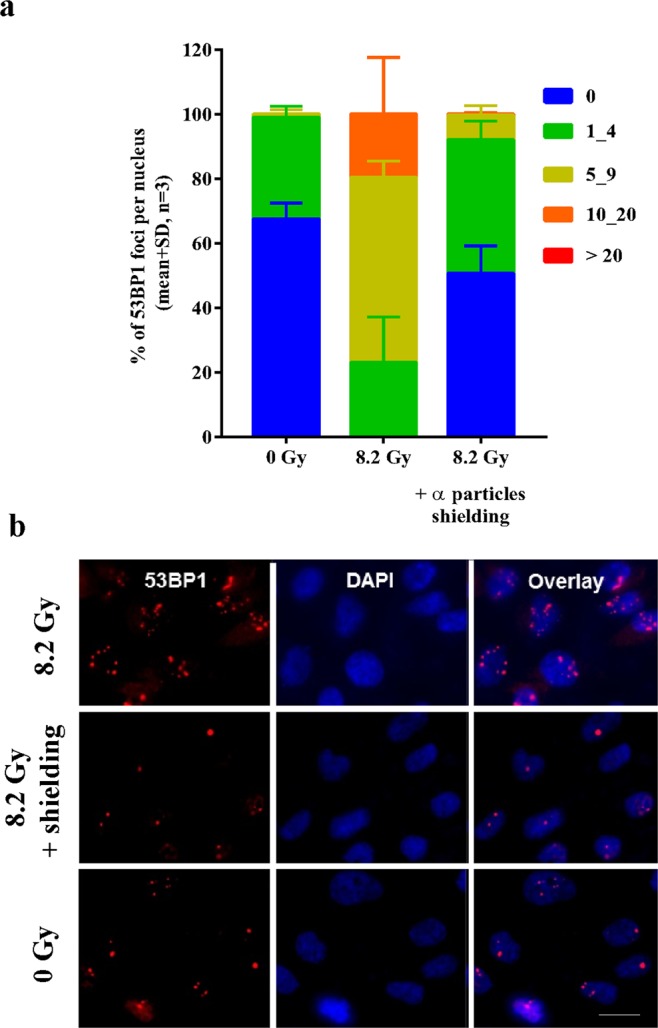
Ra-223 induced damage via α-radiation. ES-2 cells were irradiated at 1.3 kBq/cm^2^ in TWs for 4 h ± α-particles shielding by using 100 μm thick folia. (**a**) The quantification of 53BP1 foci to indicate DSBs per nucleus in percentage after αβγ- and βγ-radiation at 1.3 kBq/cm^2^. (**b**) The immunofluorescence images of 53BP1 (*red*) and DAPI (*blue*) after α-radiation ±100 μm thick folia at 4 h. Scale bar is 12 μm.

Ra-223 emits 95.3% α-particles, along with small amounts of β- and γ-radiation. In order to validate our method and to verify that α-particles will reach the cells, ES-2 cells were irradiated in TWs with 1.3 kBq/cm^2^ activity for 4 h, giving a dose of 8.2 Gy. To distinguish radiation damage caused by βγ-radiation, 100 µm thick folia, which absorbed all α-particles, was placed on top of the radiation source in control wells to shield the TWs from α-radiation. After the exposure time, cells were fixed and stained with 53BP1 antibody to detect DNA double-strand breaks (DSBs). Preliminary studies applying a double-staining with γH2AX and 53BP1 after α-radiation showed a similar foci distribution per nuclei for each staining method. Because it is independent from the replication of DSBs 53BP1 staining was used for DNA DSBs detection for the remaining experiments (Fig. S1). The nuclei with 53BP1 foci were classified into 5 groups: (i) nuclei without any foci; (ii) nuclei; with 1 to 4 foci; (iii) 5 to 9 foci; (iv) 10 to 20 foci and; (v) >20 foci per nuclei. The results showed that the cells without α-particles shielding had a higher number of DSBs (20% of a 10_20 foci per nuclei group) as compared to cells with folia shielding (Fig. 3a). No α-radiation-mediated 53BP1 foci per nuclei were observed if the Ra-223 source was shielded with folia (Fig. 3b). These results showed that the cells in TWs were irradiated mostly with α-particles from Ra-223. These data indicate that the TW system is suitable for studying α-particle specific effects on tumor cell lines *in vitro*.

### DNA DSB damage is increased by time and activities of α-radiation in various tumor cell lines

**Figure 4:**
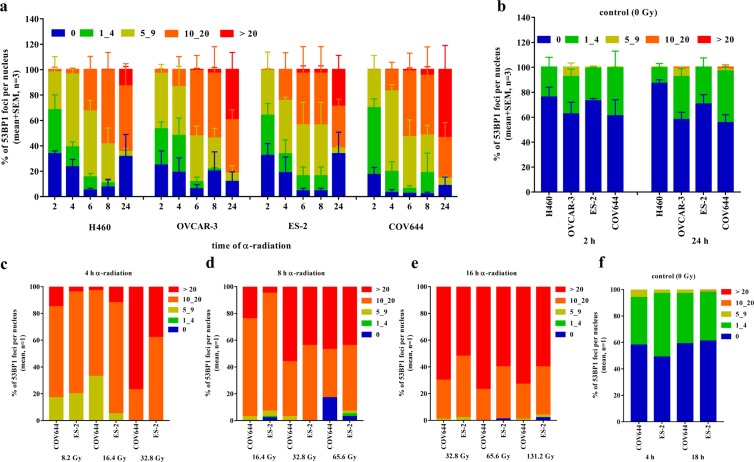
DNA damage after prolonged α-radiation in tumor cells lines. (**a**) The quantification of 53BP1 foci per nucleus in percentage at different time points after α-radiation of 1.3 kBq/cm^2^. (**b**) Un-irradiated controls. (**c**,**d**) DNA damage after α-radiation in ovarian cancer cells lines. The quantification of 53BP1 foci per nucleus in percentage at different time points after α-radiation of 4, 8, 16 hours. (**f**) un-irradiated controls.

To prove our hypothesis that the amount of an initial DNA damage will increase with time, H460, OVCAR-3, ES-2 and COV644 cells were irradiated with the activity of 1.3 kBq/cm^2^ for 2, 4, 6, 8 and 24 h, giving doses of 4.1, 8.2, 12.3, 16.4 and 49.2 Gy, respectively. The cells were then fixed and stained with 53PB1 for an identification of DNA damage by detecting DSBs (clustered double strand breaks). As shown in Fig. 4a, the amount of DNA DSBs increased in a time-dependent manner in all cell lines. However, all cell lines showed an increasing number of undamaged nuclei starting at the 8 h time point. These seemingly contradictory results might be explained either by DNA repair or by the dilution of radiation-induced damage with new undamaged cells by cell division (Fig. S2).

To evaluate the contribution of variable activities of Ra-223 with constant time, COV644 and ES-2 cells were irradiated with activities of 1.3, 2.6 and 5.3 kBq/cm^2^ for 4, 6 or 16 h, giving doses of 8.2, 16.4, 32.8 Gy for 4 h, 16.4, 32.8, 65.6 Gy for 8 h and 32.8, 65.6 and 131.2 Gy for 16 h exposure, respectively. DNA damage was evaluated by 53BP1 foci counting. The results showed (Fig. 4c–e) that the percentages of cells, which contain more than 20 foci per nuclei, increased at the low activity dose of 1.3 kBq/cm^2^ from 4 h to 16 h for all cells tested, e.g. from 15% to 70% in COV644 cells and from 4% to 60% in ES-2 cells. If the higher radioactive dose of 5.3 kBq/cm^2^ was used, in the investigated cell lines the percentages of cells, which contained more than 20 foci per nuclei, showed a more variable picture. E.g. in COV644 cells the percentage of foci >20 remained constant from 4 h to 16 h at 77% to 73% whereas in ES-2 cells, they increased from 38% to 60%. This meant, that for short exposure times higher activity of Ra-223 induced more DNA damage as compared to lower activity. In contrast to this is a longer exposure time of 16 h, where this difference was no longer observed.

### Absorbed dose as well as exposure time reduced cell survival

**Figure 5:**
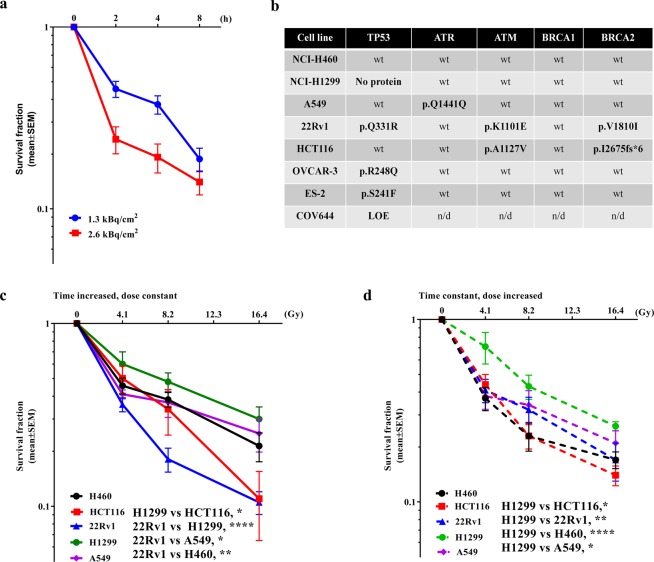
The radiosensitivity of cancer cells after α-radiation from Ra-223. (**a**) Cell survival after 2, 4, 8 and 24 h α-radiation with the activity of 1.3 and 2.6 kBq/cm^2^ in H460, n = 2. (**b**) The table of DNA mutation background in several cancer cell lines. (**c**) Survival fraction after α-radiation with the activity of 1.3 kBq/cm^2^ for 2, 4 and 8 hours, n ≥ 2. (**d**) Survival fraction after α-radiation with the activities of 1.3, 2.6, 5.3 kBq/cm^2^ for 2 hours, n = 4. Giving absorbed doses of 4.1, 8.2 and 16.4 Gy.

Using α-radiation the absorbed dose can be delivered to the tumor cells in a very short time within a few seconds (data not shown). As the above experiments have shown, the absorbed dose not only depends on the amount of the radioactivity used but also on the exposure time. In order to investigate these parameters with respect to long-term effects on cell survival, the human non-small cell lung cancer H460 were used, as they show high radioresistance in radiotherapy^12,13^. The H460 cells were exposed to Ra-223 activity at activities of 1.3 and 2.6 kBq/cm^2^ for 2, 4, and 8 h and cell survival was evaluated by colony formation assay. The data showed that cell survival in H460 cells was decreased from 46% to 19% and from 24% to 14% by increasing the exposure time from 2 to 8 hours as well as by increasing the amount of radioactivity from1.3 and 2.6 kBq/cm^2^ (Fig. 5a), e.g. 46% to 24% survival fraction (2 h). Following the hypothesis that DNA repair could play an important role in cell survival during prolonged radiation, H460, H1299, A549, HCT116 and 22Rv1 cell lines with different DNA mutation status (see Fig. 5b for details) were irradiated with 1.3 kBq/cm^2^ for 2, 4, and 8 h. The results with respect to DNA mutation status demonstrated that if H460, A459, H1299, HCT116, 22Rv1 cell lines underwent prolonged irradiation, the survival fraction seemed to be different for each cell line (Fig. 5c). The H1299, A549 and H460 cells showed the lowest radiosensitivity with a survival fraction of 21–30% after 8 h. HCT116, 22Rv1 showed only 11% surviving cells after 8 h (Fig. 5c). To investigate the effect of different activities with constant time exposure to α-radiation on cell survival, the same cell lines were irradiated with the activity of 1.3, 2.6, 5.3 kBq/cm^2^ for 2 h, giving absorbed doses of 4.1, 8.2 and 16.4 Gy, respectively. As shown in Fig. 5d, H1299 cells are still more radioresistant than the other cell lines but the variability of cell survival between the cell lines was reduced.

### α-radiation caused cell cycle arrest

**Figure 6:**
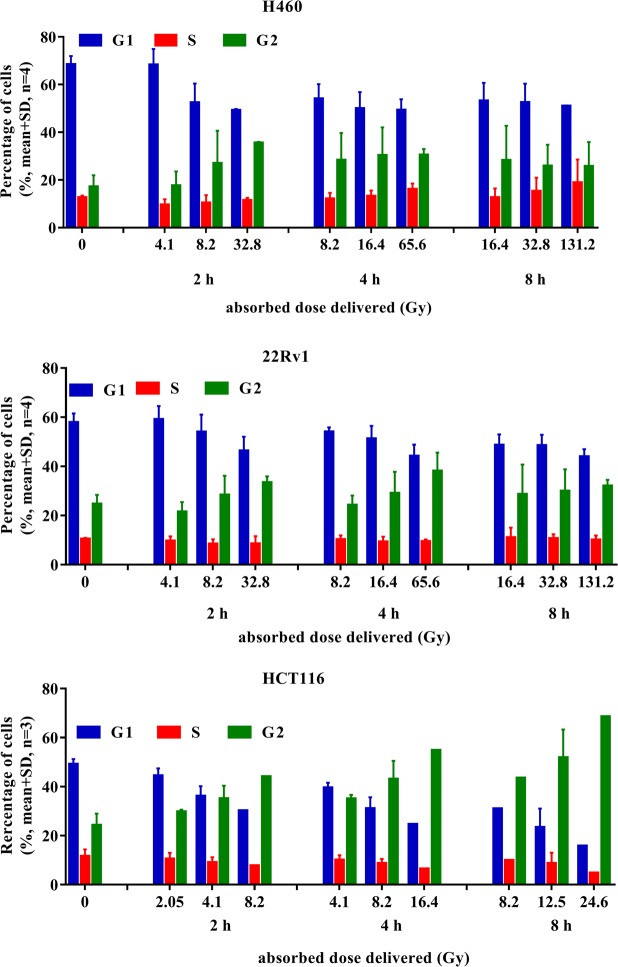
The cell cycle distribution after α-radiation in tumor cell lines.

One of the possible responses of the cell to DNA damage is cell cycle arrest in order to allow time for DNA repair. Specific radiation-induced checkpoints are activated leading mostly to G_2_/M cell phase arrest. The G_2_/M checkpoint is activated only in response to a level of 10-20 DSBs^14^. Several cell lines were investigated to see if the cells get sufficient DNA damage that they induce cell cycle arrest after radiation. H460, 22Rv1, HCT116 cell lines were exposed to α-radiation with the activities of 2.6, 5.3 and 10.5 kBq/cm^2^ for 2, 4, and 8 h, giving doses of 4.1, 8.2, 32.8 Gy for 2 h, 8.2, 16.4, 65.6 Gy for 4 h and 16.4, 32.8 and 131.2 Gy for 8 h exposure and for HCT116 2.05, 4.1, 8.2 for 2 h, 4.1, 8.2, 16.4 for 4 h and 8.2, 16.4, 24.8 for 8 h. The results showed that α-radiation induced G_2_/M arrest also in a dose-dependent manner and reached the maximum at 2 h of irradiation from 17% to 35% in H460, at 4 h from 24% to 38% in 22Rv1 and at 8 h from 24% to 68.5% in HCT116. It seems that after prolonged α-radiation the percentage of cells in G_2_/M reached the maximum (Fig. 6). HCT116 cells were clearly the most radiosensitive. Giving an absorbed dose of 4.1 Gy for 2 h led to a reduced number of cells in G1 and an accumulated of cells in G_2_/M cell phase. Overall, α-radiation induced G_2_/M arrest in H460, 22Rv1 and HCT116 cell lines, which can lead to induction of cell death.

### The detection of DNA damage in single cells after α-radiation

**Figure 7:**
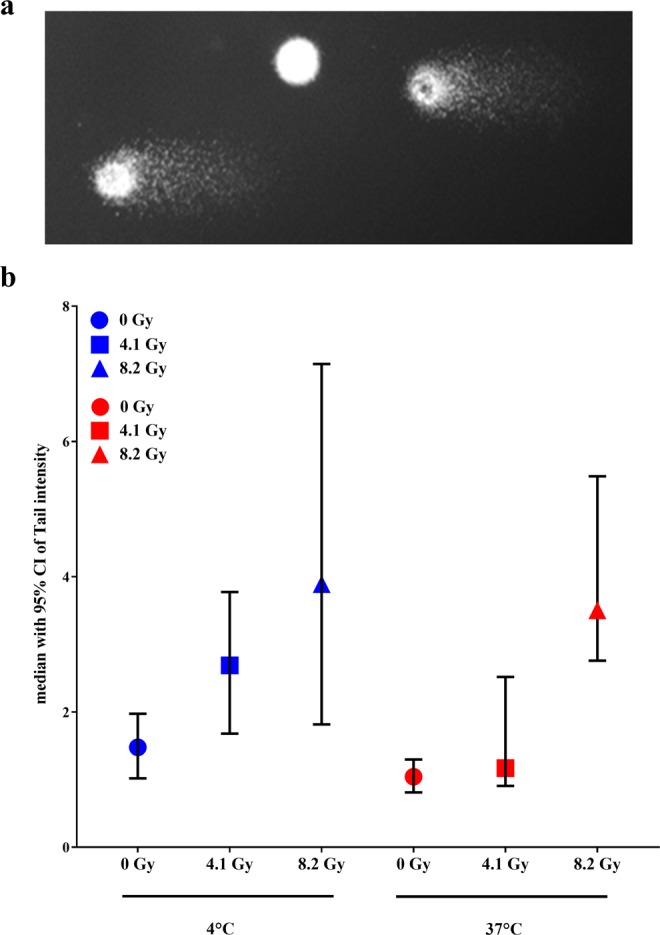
The detection of DNA damage in H460. (**a**) The comets after the electrophoresis. (**b**) The median of tail intensity after α-radiation with absorbed doses of 4.1 and 8.2 Gy for 1 h at 4 °C (*blue symbols*) and at 37 °C (*red symbols*).

It has been reported that DNA repair mechanisms start immediately after the nuclei has received the DNA damage under standard conditions^15,16^. We wanted to answer the question if DNA damage can be partially repaired during prolonged radiation and if this could be the answer to the observed difference between increased activity and increased radiation time observed above. In order to prevent DNA repair, cells were irradiated at 37 °C (optimal activity of DNA repair enzymes), with activities of 2.6 and 5.3 kBq/cm^2^ for 1 h, giving the dose of 4.1 and 8.2 Gy (low activity of DNA repair enzymes), respectively, followed by evaluation of primary DNA fragmentation using the comet assay. The results from the comet assay demonstrated that α-radiation induced DNA fragmentation in a dose-dependent manner under both conditions. The amount of DNA damage shown as tail intensity was lower with 4.1 Gy at 37 °C when compared to the cells irradiated at 4 °C. However, we did not detect the difference in DNA damage between both temperature conditions at 8.2 Gy (Fig. 7b). This could indicate that at a low amount of damage the cells were able to repair the damage at least when incubated at 37 °C whereas 4 °C incubation prevented the DNA repair mechanisms. During data evaluation, we observed that not all nuclei showed DNA damage after α-radiation, as some of the nuclei showed no tail formation (Fig. 7a).

## Discussion

In the present study, we found that the Transwell system is a useful tool to study α-particle specific effects on tumor cell lines *in vitro*. Due to the fact that the α-particles from Ra-223 hit the cells with hardly any absorption in medium, we could investigate the direct radiobiological effects of α-radiation on various tumor cells in terms of cell survival, cell cycle arrest, direct comet assay and indirect detection of DNA damage by immunofluorescent staining, and also calculate the absorbed doses by Monte-Carlo simulation.

The dosimetry of α-emitters is not a complex issue and it has been addressed in a large number of publications. Firstly, we proceeded from the fact that the nucleus is more radiosensitive to α-radiation than cytoplasm^17^. We focused our calculation of absorbed dose on the nuclei and the hits distribution from Ra-223. We showed that the cell survival was reduced in all tumor cell lines via cluster DNA damage due to the high number of hits in the nuclei: 24.7 ± 5 (2 h) under our conditions. Moreover, we found that the DNA damage intensified with increasing the time of α-radiation or increasing the amount of α-particles. Unexpectedly, we observed that by prolonging the irradiation time the percentage of the cells without DNA damage increased. This suggests that either the number of hits is not enough to kill the cells or some DNA damage was repaired or the cells might die later via induced senescence. Several publications propose variable hit rates of 1 to 25 α-particles are required to induce lethal damage to cells^18–21^.

We hypothesize that if the cells were exposed longer to α-radiation or to a higher activity of Ra-223 then the possibility to hit more cells increases. Our findings demonstrated that cell survival decreased as the activity increases. This means that the cells exposed to higher activity thereby a higher number of hits occurred, which induced unrepaired clustered DNA damage and therefore, the ability to form colonies is reduced. The most important finding in this study was that the tumor cells demonstrated a broad variability in radiosensitivity after prolonged α-radiation, this could potential depend on the cell proliferation rates^22^. High-LET radiation induces DNA damage by direct ionizations of the DNA molecule and leads to a large energy deposition, resulting in the induction of clustered DNA damage (also termed multiple damage sites)^23,24^. Repair of complex DNA lesions induced by high-LET radiation is still not well understood. DSBs can be repaired at least by three well-known pathways - homologous recombination (HR), non-homologous end joining (NHEJ), and alternative end joining. It has been proposed that complex DSBs generated by high LET irradiation are repaired by HR and not NHEJ in mammalian cells^25^. However, recent reports have indicated that NHEJ may play a prominent role in repair of carbon ion-induced damage^26,27^. Our studies suggest that after prolonged exposure to α-radiation, the DNA mutation status (*BRCA1/2, DNA-PK, ATM, ATR, TP53*, *PIK3CA*, *PTEN* and etc.) might play an important role in the radioresistance of various tumor cells. We observed that 22Rv1 and HCT116 cells with DNA mutation in *ATM* and *BRCA2* were more radiosensitive than H460 (*TP53* wt) and H1299 (*TP53* mut) cells. Although the impact of genetic alterations on radioresistance seems to diminish in tumor cells if the cells were irradiated for a short time. In line with the results from the direct DNA damage detection by the comet assay, we demonstrated that inhibition of DNA repair mechanisms by low temperature led to higher amounts of radiation–induced DNA damage as compared to cells at 37 °C. This effect was observed at low activity of Ra-223, but not at higher activity. This may suggest that DNA repair mechanisms may reach a limit at higher Ra-223 activities, mechanistically pointing to a non-linear dose-response.

In addition, we investigated the cell cycle progression as a DNA-damage response to α-radiation. In the case of DNA damage, the cell cycle progression was inhibited by a complex network of signaling and transducing pathways^28^. This cell cycle arrest provided cells with additional time to repair the DNA damage before progressing into the next cell cycle phase. Very early studies have demonstrated that ionizing radiation induced cell cycle arrest in S- and G_2_/M-phase^29^ and this was also the case after α-radiation^30,31^. It is important to know that the G2/M checkpoint can be activated in response to a level of 10–20 DSBs. As the G2/M checkpoint was insensitive below 10–20 DSBs, cells underwent mitosis with unrepaired DSBs which could result in loss of genetic material^14^. We observed α-radiation induced cell cycle arrest in G_2_/M phase in a dose-dependent manner after short time α-radiation (2 h) and reached the plateau by prolonged exposure to α-radiation.

## Conclusion

We demonstrated that the Transwell system is suitable for the assessment of α-radiation-mediated effects in tumor cells *in vitro*. Based on a single set of parameters, we showed: (i) alpha mediated DNA DSB upon short-term exposure in contrast to background; (ii) induction of DSB was cell line independent but dose-and time-dependent; (iii) short-term exposure led to an inhibition of the surviving fractions in various tumor cell lines. These finding encourage us to further investigate the impact to DNA repair in the cell survival upon α-radiation in various tumor cell lines.

## Supplementary information

Supplementary Data
