# Supplementary material for: Radiobiological effects of the alpha emitter Ra-223 on tumor cells
Source: Sci Rep. 2019 Dec 6;9:18489. doi: 10.1038/s41598-019-54884-7 (PMC6898438; doi:10.1038/s41598-019-54884-7)
Supplement: Supplementary file 1 — Supplementary Data [file 41598_2019_54884_MOESM1_ESM.docx]

**Radiobiological effects of the alpha emitter Ra-223 on tumor cells**

Kristina Bannik^1^, Balázs Madas^2^, Marco Jarzombek^1^, Andreas Sutter^1^, Gerhard Siemeister^1^, Dominik Mumberg^1^, Sabine Zitzmann-Kolbe^1^

^1^ Bayer AG, Pharmaceuticals Division, Berlin, Germany

^2^ MTA Centre for Energy Research, Budapest, Hungary

**Supplemental material**

**Supplemental figure 1**

**
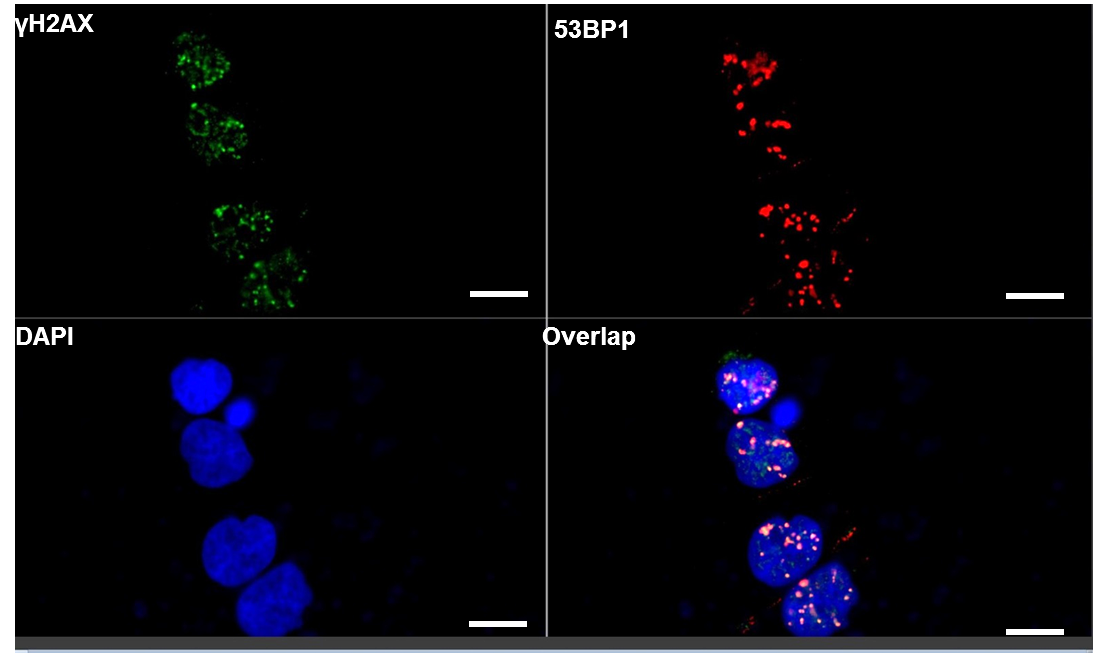
**

**SUPPLEMENTAL FIGURE 1** The immunofluorescence images of H460 lung cancer line. The cells were α-irradiated with activity of 1.3 kBq/cm^2^ for 8 h. γH2AX (green), 53BP1 (red) and DAPI (blue). Scale bar is 15 μm

**Supplemental figure 2**

**
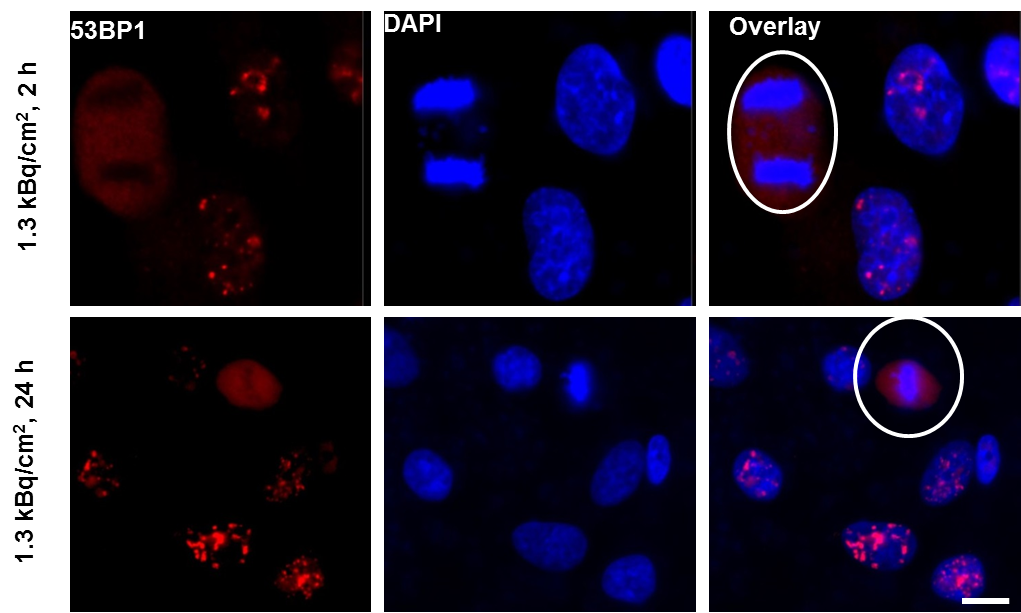
**

**SUPPLEMENTAL FIGURE 2** The immunofluorescence images of ES-2 ovarian cancer line. The cells were α-irradiated with activity of 1.3 kBq/cm^2^ for 2 and 24 h. 53BP1 (red) and DAPI (blue). The circles represent mitotic cells. Scale bar is 12 μm
